# Antibacterial activity of vB_AbaM_PhT2 phage hydrophobic amino acid fusion endolysin, combined with colistin against *Acinetobacter baumannii*

**DOI:** 10.1038/s41598-023-33822-8

**Published:** 2023-05-08

**Authors:** Sutthirat Sitthisak, Suphattra Manrueang, Supat Khongfak, Udomluk Leungtongkam, Rapee Thummeepak, Aunchalee Thanwisai, Nathan Burton, Gurneet K. Dhanoa, Panagiotis Tsapras, Antonia P. Sagona

**Affiliations:** 1 Department of Microbiology and Parasitology, Faculty of Medical Science, Naresuan University, Muang, Phitsanulok, 65000 Thailand; 2 Centre of Excellence in Medical Biotechnology, Faculty of Medical Science, Naresuan University, Phitsanulok, 65000 Thailand; 3 School of Life Sciences, University of Warwick, Coventry, CV4 7AL UK

**Keywords:** Phage biology, Bacteriophages

## Abstract

Phage lytic enzymes are promising antimicrobial agents. In this study, an endolysin derived from vB_AbaM_PhT2 (vPhT2), was identified. This endolysin represented the conserved lysozyme domain. Recombinant endolysin (lysAB- vT2) and hydrophobic fusion endolysin (lysAB-vT2-fusion) were expressed and purified. Both endolysins showed lytic activity against bacterial crude cell wall of Gram-negative bacteria. The MIC of lysAB-vT2-fusion was 2 mg/ml corresponding to 100 µM, while the MIC of lysAB-vT2 was more than 10 mg/ml (400 µM). Combination of lysAB-vT2-fusion with colistin, polymyxin B or copper was synergistic against *A. baumannii* (FICI value as 0.25). Antibacterial activity of lysAB-vT2-fusion plus colistin at the fractional inhibitory concentrations (FICs) revealed that it can inhibit *Escherichia coli*, *Klebsiella pneumoniae* and various strains of extremely drug-resistant *A. baumannii* (XDRAB) and phage resistant *A. baumannii*. The lysAB- vT2-fusion still retained its antibacterial activity after incubating the enzyme at 4, 20, 40 and 60 °C for 30 min. The lysAB-vT2-fusion could inhibit the mature biofilm, and incubation of lysAB-vT2-fusion with T24 human cells infected with *A. baumannii* led to a partial reduction of LDH release from T24 cells. In summary, our study highlights the antimicrobial ability of engineered lysAB-vT2-fusion endolysin, which can be applied for the control of *A. baumannii* infection.

## Introduction

*Acinetobacter baumannii* is a Gram-negative bacterium emerging as a major cause of nosocomial infection and is particularly problematic for patients on ventilator support with long term hospital stays. Associations with an increasing incidence of pan drug-resistant *A. baumannii*, which resist treatment to the last resort antibiotic colistin, have fueled the research of novel antimicrobial agents. One potential candidate is phage therapy, which uses lytic bacteriophages or phage enzymes to treat bacterial infections that are resistant to antibiotics^1,2^. However, there are limitations to using phage virions, including phage specificity for a narrow host range and the potential for resistance development^3^. Our previous study showed that 50% of *A. baumannii* isolates in Thailand were phage resistance strains against 17 lytic *A. baumannii* phages tested^4^. Thus, there is increasing interest in using bacteriophage lytic enzymes, or endolysins, as antimicrobial agents compared to using phage virions. Endolysins are phage enzymes that function by hydrolyzing the peptidoglycan layer, resulting in a sudden drop in turgor pressure and osmotic lysis, which causes bacterial cell death^5^. Recombinant endolysins from various *A. baumannii* phages have been cloned, expressed, and characterized^6–14^. Yuan et al. and Kim et al. showed that endolysins, Abtn-4 and LysSS, exhibited broad antimicrobial activity against several Gram-positive and Gram-negative bacteria. In addition, endolysins LysAB3, Abtn-4, and Abp013 were observed to be effective in reducing *A. baumannii* biofilm^9,13,14^. The activity of endolysins such as LysABP-01, ABgp46, LysMK34, and ElyA1 can be enhanced by combining them with outer membrane permeabilizers, such as organic acids^7^ and colistin^8,10,11^. In addition, engineered endolysins have been shown to have improved antibacterial activity against colistin-resistant *A. baumannii* strains^15^.

In a previous study, we identified vB_AbaM_PhT2 which showed best efficacy against its host and various *A. baumannii* strains. This bacteriophage also showed synergy with colistin^16^. A genomic analysis of vB_AbaM_PhT2 was done using Next Generation Sequencing (NGS) and allowed for the identification of putative genes encoding lysin. These enzymes hydrolyze the peptidoglycan in bacterial cell walls and therefore are good candidates as antibiotic alternatives. Although, many endolysins in *A. baumannii* have been characterized, there are fewer studies on the role of hydrophobic amino acid fusion endolysin. The lysis ability of *E. coli* endolysin was enhanced by the addition of a hydrophobic amino acid at the C-terminus of the endolysin^17^. Therefore, we aimed to investigate the antimicrobial activity of recombinant phage endolysins and hydrophobic fusion- endolysins and their potential synergy with antibiotics, disinfectants, heavy metals and phage. Fusion-endolysins showed high lytic activity compared to native enzyme. Cytotoxicity assays, biofilm forming inhibition and antimicrobial activity against various strains of *A. baumannii* isolates from various Thai hospitals of fusion- endolysins were investigated.

## Results

### Characterization of the recombinant phage-derived endolysin (lysAB-vT2) and the phage endolysin fusion (lysAB-vT2-fusion)

**Figure 1:**
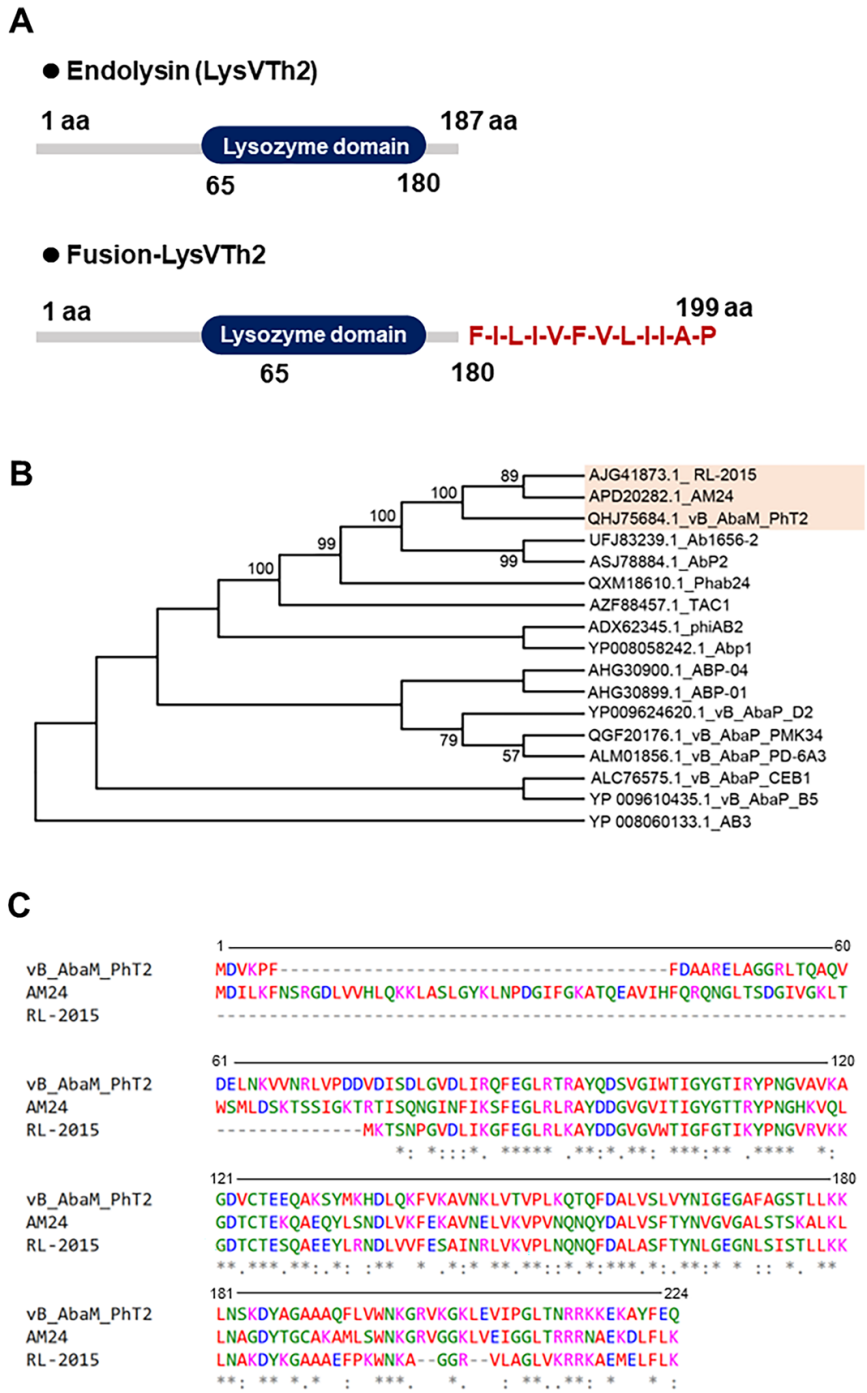
Characterization and bioinformatic analysis of endolysin LysVTh2 (lysAB-vT2). (**A**) Schematic diagram of the conserved domain of putative endolysin (LysVTh2, lysAB-vT2) and fusion-LysVTh2 (lysAB-vT2-fusion). This diagram was predicted using the Pfam webserver. (**B**) Bootstrap consensus tree showing the phylogenetic relationship between LysVTh2 (lysAB-vT2) and previously reported 16 of *A. baumannii* lysins. The evolutionary history was inferred using the Neighbor-Joining method with 1,000 bootstrap replications and was conducted in MEGA 11. (**C**) Comparison analysis of vB_AbaM_PhT2 (LysVTh2, lysAB-vT2), AM24 (Accession no. APD20282.1) and RL-2015 (Accession no. AJG41873.1) endolysins using the multiple sequence comparison by Clustal Omega algorithm.

We investigated the genome of phage vB_AbaM_PhT2 (vPhT2) (MN864865) to identify endolysin gene. The endolysin has been detected (QHJ75684.1) in the genome of the phage vPhT2. The lysAB-vT2 contains 187 amino acid residues, while lysAB-vT2-fusion contains 199 amino acid residues (Fig. 1A). A lysozyme conserved domain was detected from amino acids 65 to 180 (Fig. 1A). Sequence of vPhT2 endolysin gene yields 53.53–60.54% sequence identity to the endolysin gene of phage RL_2015 (Accession no. AJG41873.1) and phage AM24 (Accession no. APD20282.1) (Fig. 1B). Comparison of amino acid sequence of lysAB-vT2 with endolysin from phage RL_2015 and phage AM24 was shown in Fig. 1C.

**Figure 2:**
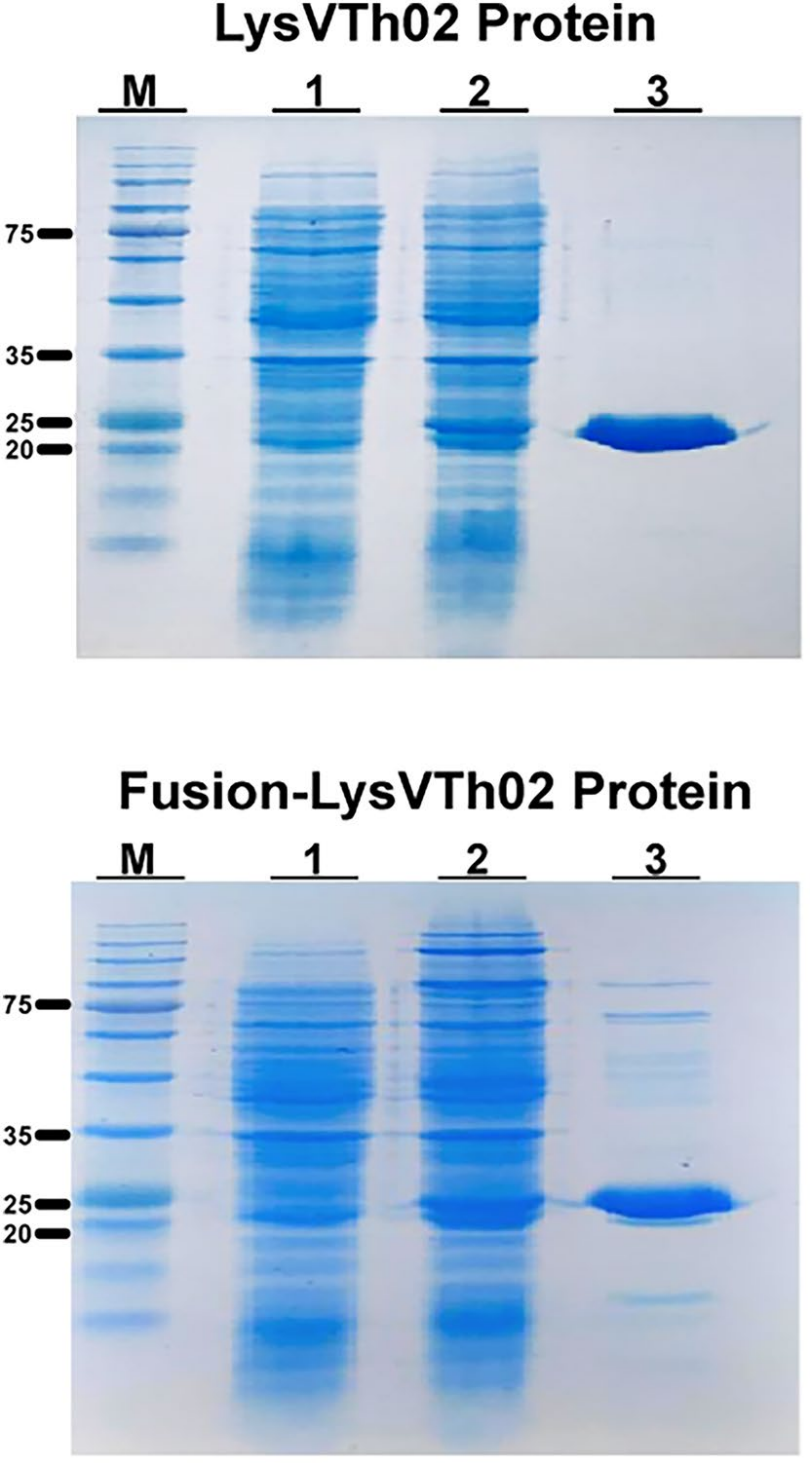
Protein expression analysis and plate lysis assay of endolysin (LysVTh2, lysAB-vT2) and fusion-LysVTh2 (lysAB-vT2-fusion). SDS-PAGE analysis for expression and purification of (LysVTh2, lysAB-vT2) and fusion-LysVTh2 (lysAB-vT2-fusion). Lane M, BLUeye prestained protein ladder; Lanes 1, un-induced bacterial lysate; Lane 2, IPTG-induced bacterial lysate; Lane 3, the purified endolysin (LysVTh2, lysAB-vT2) and fusion-LysVTh2 (lysAB-vT2-fusion) after dialysis.

Both lysAB-vT2 and lysAB-vT2-fusion were expressed in soluble form. Both purified recombinant protein yields were 5.16 and 4.44 mg per liter of culture. The purified proteins were examined by SDS–PAGE and bands of approximately 25 kDa were observed, which corresponds to the predicted molecular mass of 25 kDa (Fig. 2).

### Lysis activity of lysAB-vT2 and lysAB-vT2-fusion against bacterial crude cells of Gram-negative bacteria

**Figure 3:**
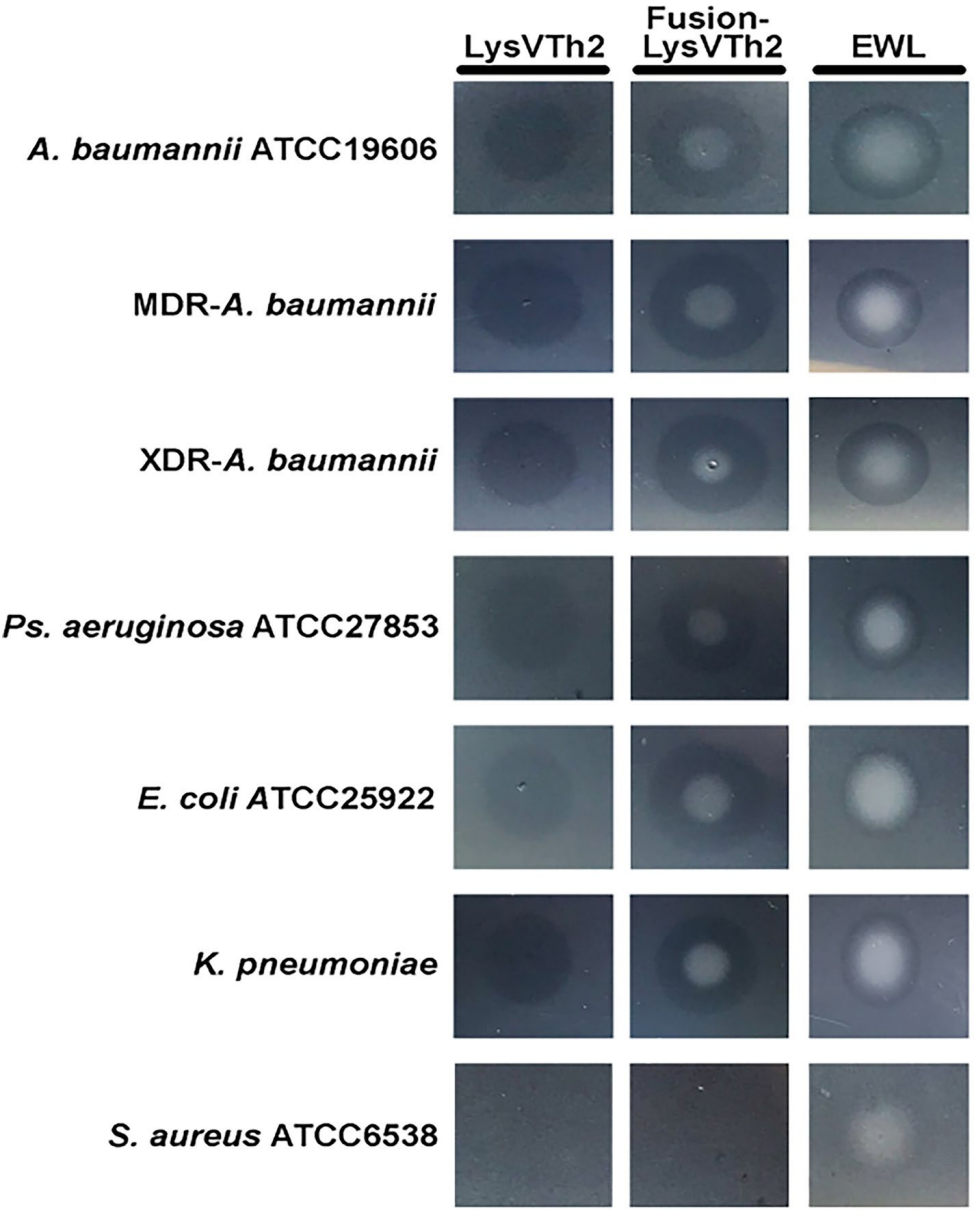
Plate lysis assay of the purified endolysin (LysVTh2, lysAB-vT2) and fusion-LysVTh2 (lysAB-vT2-fusion) against *A. baumannii.* The plate lysis assay of the purified endolysin (LysVTh2, lysAB-vT2) and fusion-LysVTh2 (lysAB-vT2-fusion) against *A. baumannii* was performed using autoclaved cells of *A. baumannii* ATCC 19606, *A. baumannii* AB003 (MDRAB), *A. baumannii* AB329 (XDRAB), *Ps. aeruginosa* ATCC 27853, *E. coli* ATCC 25922, *K. pneumoniae* ATCC 27736 and *S. aureus* ATCC 6538. Egg white lysozyme (EWL) was used as positive control.

The recombinant protein was utilized in a plate-spot assay to determine the lytic activity of both endolysins against bacterial crude cells. The results showed that both proteins had lytic activity towards *A. baumannii* strains ATCC 19606, MDRAB and XDRAB strain (Fig. 3). They also have lytic activity against other Gram-negative crude cells, such as *E. coli, P. aeruginosa* and *K. pneumoniae* (Fig. 3). However, lytic activity was not detected in the gram-positive strain *S. aureus*. The inhibition zone of Purified fusion- LysVTh2 was larger than the purified LysVTh2 and the egg white lysozyme (Table S1).

### Minimum inhibitory concentration (MIC) of lysAB-vT2 or lysAB-vT2-fusion

We compared efficacy of two recombinant endolysin by determined the MIC. The MIC of purified lysAB-vT2 or lysAB-vT2-fusion was tested using bacterial growth inhibition assays for its ability to inhibit the growth of viable cells of *A. baumannii* ATCC 19606. The results indicated that the MIC of lysAB-vT2-fusion was 2 mg/ml corresponding to 100 µM, while the MIC of lysAB-vT2 was more than 10 mg/ml (400 μM).

### Synergism of lysAB-vT2-fusion with antibiotics, disinfectants, heavy metals and bacteriophage

**Figure 4:**
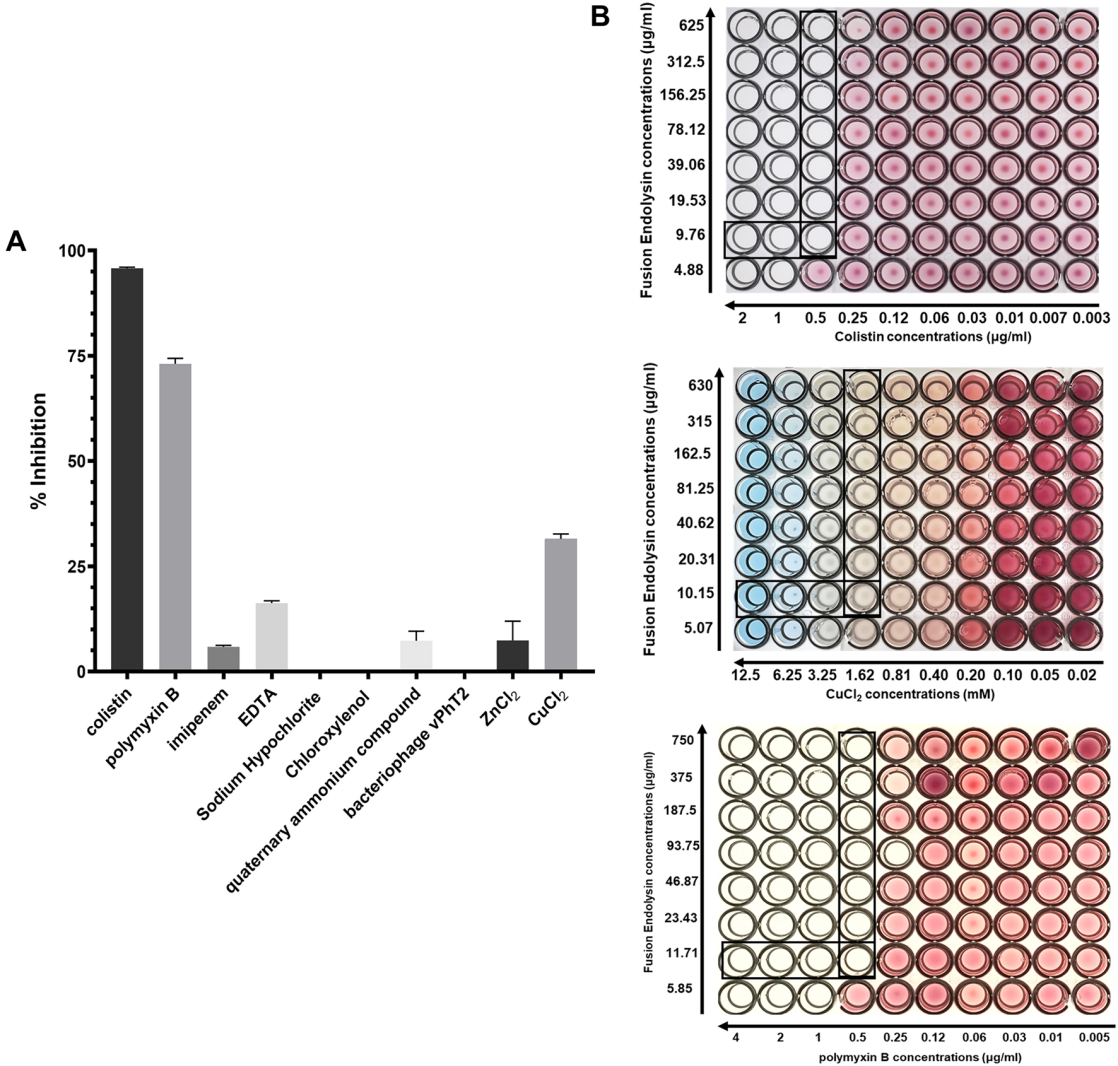
Screening and confirmatory testing for synergistic interaction and synergy measurement by checkerboard analysis. (**A**) The growth inhibition rate (the bar graph) of lysAB-vT2-fusion with three antibiotics (imipenem, colistin and polymyxin B), three disinfectants (quaternary ammonium compound, Chloroxylenol, Sodium Hypochlorite), two heavy metals (ZnCl_2_, CuCl_2_), and bacteriophage vPhT2. Data are expressed as the mean percentage ± SD of triplicate experiments. (**B**) checkerboard assay of lysAB-vT2-fusion with colistin, CuCl_2_ and polymyxin B.

We evaluated the effect of lysAB-vT2-fusion combined with antibiotics, disinfectants, heavy metals and bacteriophage vPhT2. The MICs of all tested agents are shown in Table S2. The synergistic effect of lysAB-vT2-fusion was screened by growth inhibition assay at 0.25 × the MIC of two agents. As shown in Fig. 4A, the combination of both endolysins plus colistin and polymyxin B, exhibited elevated antibacterial activity followed by CuCl_2_ with percent inhibition as 95.8, 73.1, and 31.5%, respectively (Table S3). Low interaction with EDTA (16.3%), ZnCl_2_ (7.4%), quaternary ammonium compound, (7.3%), and imipenem (5.9%) with lysAB-vT2-fusion was observed (Table S3). There was no interaction when combining lysAB-vT2-fusion with Chloroxylenol, Sodium Hypochlorite, and bacteriophage vPhT2. We performed a checkerboard assay for measuring antibiotic synergy of colistin, polymyxin B and CuCl_2_. The fractional inhibitory concentrations (FICs) for the combination of lysAB-vT2-fusion with colistin was 9.76 and 0.5 ug/ml, polymyxin B was 11.71 and 0.5 ug/ml, and CuCl_2_ was 10.15 ug/ml and 1.62 mM (Fig. 4B). The FIC indexes of lysAB-vT2-fusion and all three agents were calculated as 0.25, which indicates synergism (Fig. 4B).

### Antibacterial activity of lysAB- vT2-fusion

**Figure 5:**
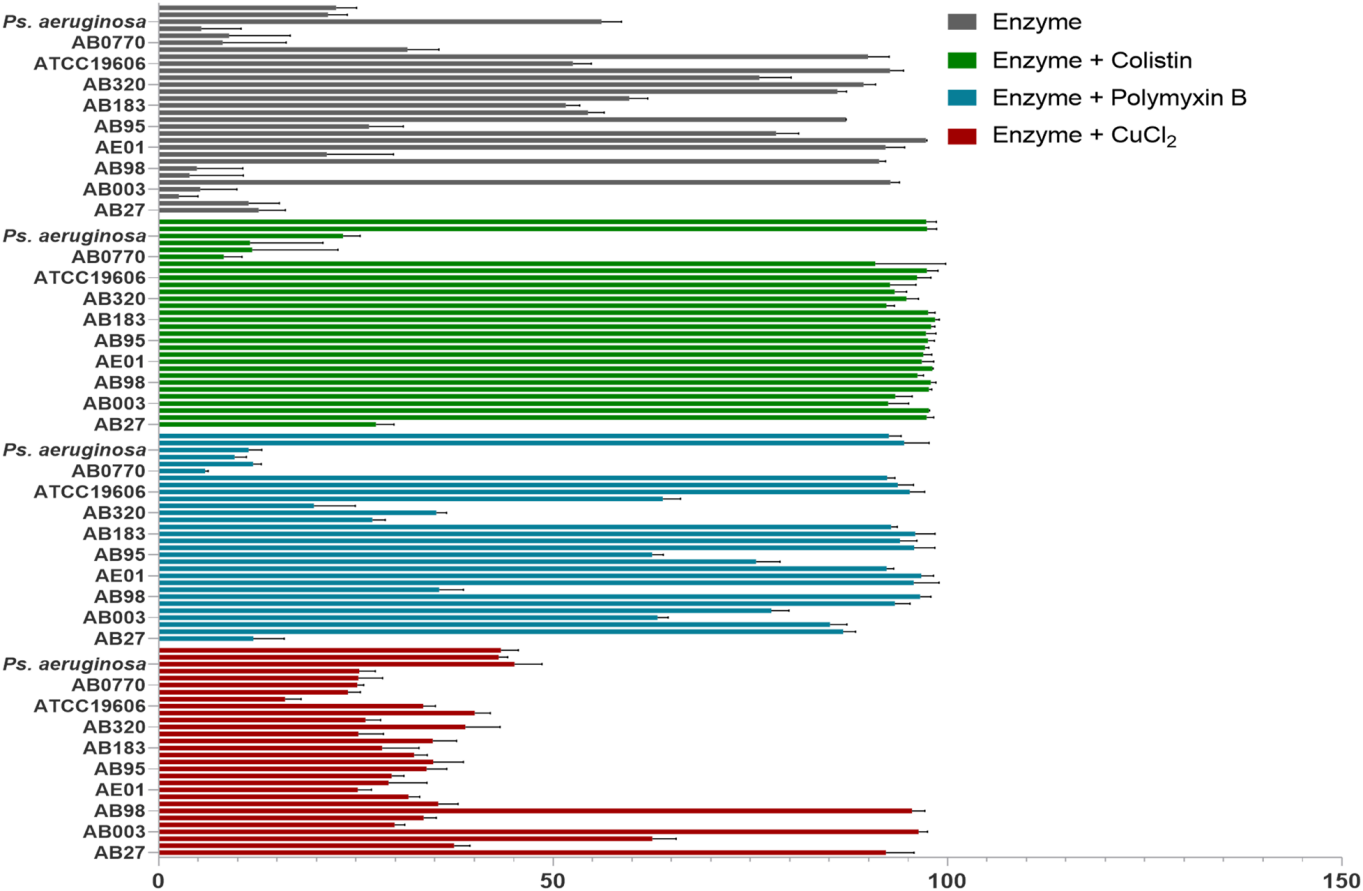
Antibacterial activity of lysAB-vT2-fusion and lysAB-vT2-fusion plus colistin, polymycin B, and CuCl_2_. Antibacterial activity of lysAB- vT2-fusion (2 mg/ml) (black bar), lysAB- vT2-fusion plus colistin (10 ug/ml + 0.5 ug/ml) (green bar), lysAB- vT2-fusion plus polymyxin B (10 ug/ml + 0.5 ug/ml) (blue bar), and lysAB- vT2-fusion plus CuCl_2_ (10 ug/ml/1.62 mM) (red bar) was determined using inhibition assay against *E.coli, P. aeruginosa, K.pneumoniae* and various strains of *A. baumannii*.

To evaluate the antimicrobial potency of lysAB- vT2-fusion, antibacterial activity of lysAB-vT2-fusion alone and lysAB-vT2-fusion in combination with colistin was determined against various strains of *A. baumannii* and other bacterial species (Table S4)*.* Among 27 *A. baumannii* strains tested, 14 strains were inhibited with 2 mg/ml enzyme with percent inhibition more than 85% (Fig. 4B and Table S5). We found increased antibacterial activity of lysAB-vT2-fusion plus colistin, which can inhibit  *E. coli* ATCC 25922, *K. pneumoniae* ATCC 27736, phage resistant *A. baumannii* MDRAB, XDRAB and NR strains. In addition, in combination with colistin, the enzyme can inhibit the growth of 22 out of 26 *A. baumannii* strains with more than 90% percent inhibition (Table S6). Enzyme alone or enzyme with colistin exhibited low inhibition of colistin resistant *A. baumannii* (Fig. 5).

### Thermal stability

**Figure 6:**
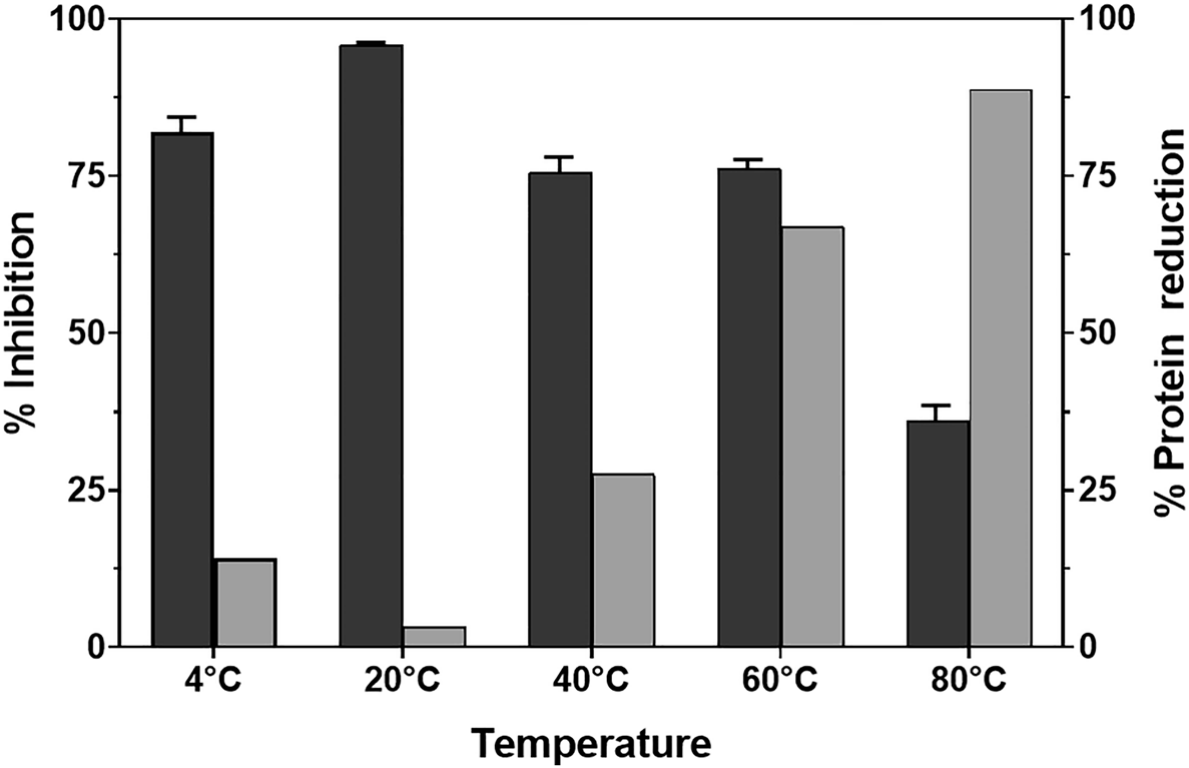
Thermal stability of lysAB-vT2-fusion. A thermal stability of antibacterial activity of lysAB- vT2-fusion was determined using bacterial inhibition assay with 2 mg/ml of lysAB-vT2-fusion after incubation at 4, 20, 40, 60 and 80 °C for 20 min. Black bar represented percent inhibition of the enzyme after thermal treatment and grey bar represented percent reduction of protein concentration.

A thermal stability test was executed to analyze the heat resistance of antibacterial activity of lysAB- vT2-fusion. Our data showed that lysAB-vT2-fusion retained antibacterial activity with the percentage inhibition at almost 95% after incubation at 20 °C. The outcomes of lysAB-vT2-fusion after incubated the enzyme at 4, 20, 40 and 60 °C showed that percent inhibitions at nearly 75%. At 80 °C, the percent inhibition of lysAB-vT2-fusion dropped to 30% (Fig. 6). Percent reduction of protein concentration was low at 4, 20, and 40 °C. We found percent reduction of protein concentration was reduced to 80% at 80 °C (Fig. 6).

### Biofilm eradication assay of lysAB-vT2-fusion and adjuvant effect with phage vPhT2

**Figure 7:**
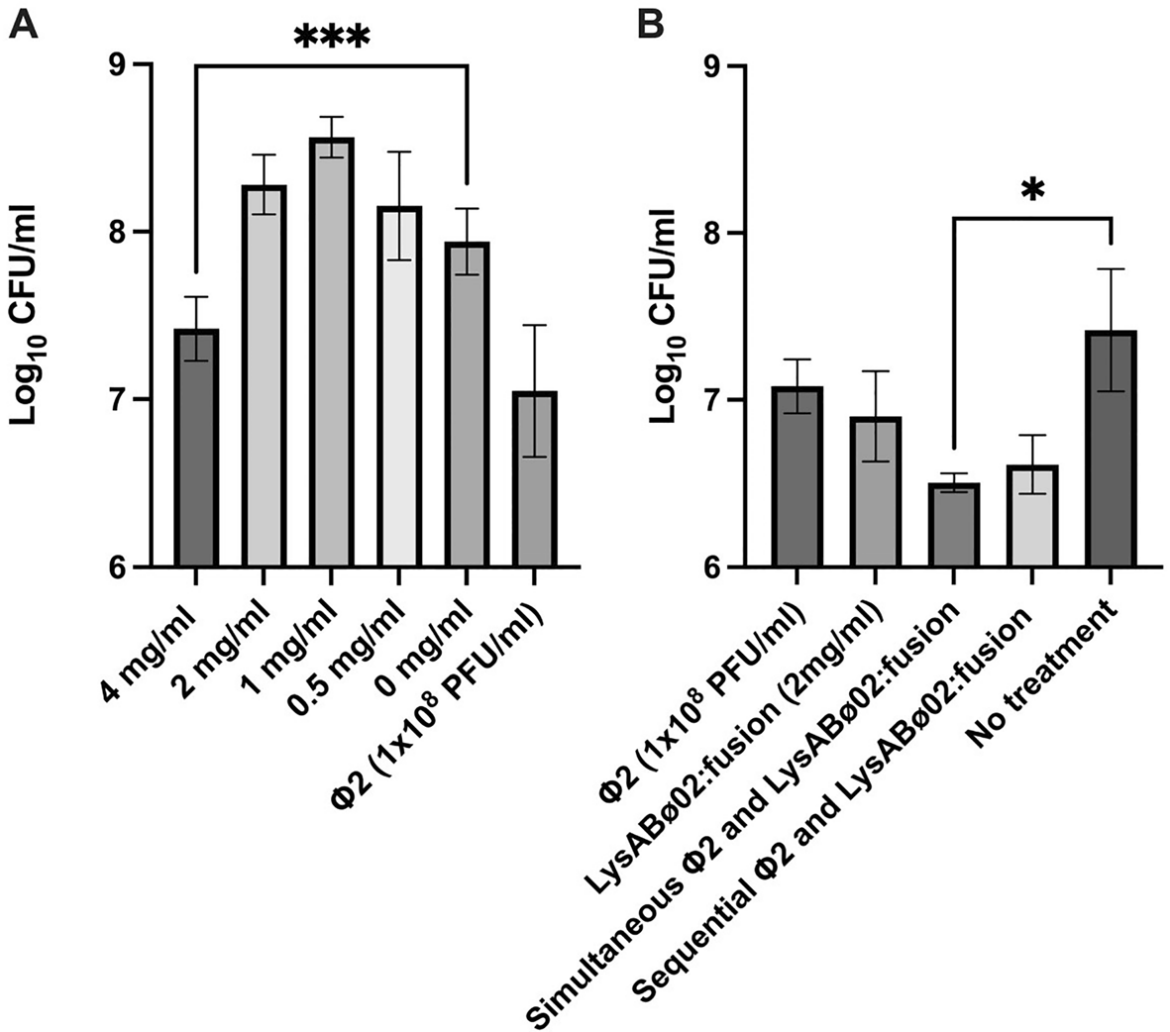
Biofilm, and cytotoxicity of lysAB-vT2-fusion. (**A**) MBEC assay quantified the disruption of mature *A. baumanni* AB183 biofilms by varying concentrations of lysAB-vT2-fusion. Biofilms of *A. baumanni* AB183 were formed on 96 well MBEC plates over 3 days and endolysin mediated killing of biofilm bacteria was quantified by a subsequent 24-h treatment using dilutions of endolysin measuring only biofilm cells. (**B**) Adjuvant effect of lysAB-vT2 fusion (2 mg/ml) with simultaneous and staggered treatment (after 24 h) of bacteriophage vPhT2 (Φ2) on mature *A. baumanni* AB183 biofilms * Asterisks denote *P* ≤ 0.05 *** Asterisks denote *P* ≤ 0.001.

Bacterial biofilm destruction and cytotoxicity of lysAB-vT2-fusion were determined to evaluate possible uses of lysAB-vT2-fusion. The biofilm assay showed that upon exposure of 4 mg/ml lysAB-vT2-fusion and 1 × 10^8^ PFU/ml of phage vPhT2 with 72-h *A. baumannii* biofilms, we found a significant reduction in CFU remaining in the biofilms. Enzyme concentrations less than 4 mg/ml increased biofilm formation (Fig. 7A).

To assess the adjuvant effect of lysAB-vT2 fusion endolysin with phage vPhT2 in the clearance of *A. baumanni* biofilms, treatment was performed by was assessed by simultaneous and sequential exposure. Our data shows that sequential treatment of endolysin for 24 h and then phage vPhT2 for another 24 h did not significantly decrease the number of CFU’s in the biofilm (Fig. 7B). However, simultaneous treatment of mature *Acinetobacter* biofilms by lysAB-vT2 fusion (2 mg/ml) and phage vPhT2 (1 × 10^8^ PFU/ml) at the same time yields significantly lower CFU remaining in the biofilms (*p* < 0.05), suggesting simultaneous treatment of endolysin and phage is better than staggered.

### Cytotoxicity of lysAB-vT2-fusion to human cells

**Figure 8:**
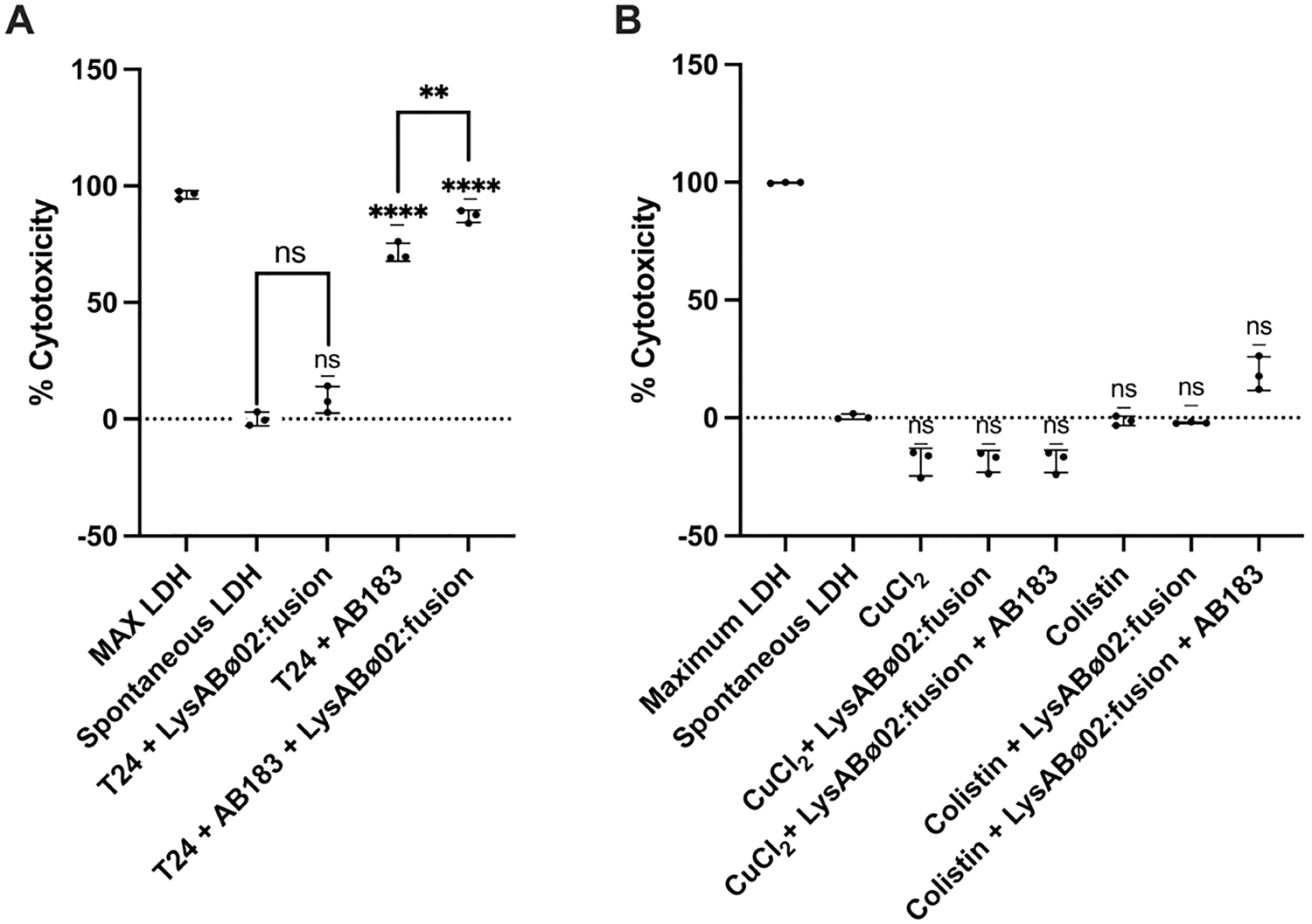
Cytotoxicity of endolysin (fusion- LysVTh2) to human epithelial cells. AB183 was grown overnight in Leibovitz medium and used to infect cells for 1 h before adding 2 mg/ml of lysAB-vT2-fusion to relevant samples and incubating for a further 23 h. LDH cytotoxicity assay was then performed using the Invitrogen CyQUANT kit. (**A**) Recovery and cytotoxicity of lysAB- vT2-fusion treatment of T24 human epithelial cells infected by *A. baumanni* AB183. (**B**) Cytotoxicity after recovery of T24 human epithelial cells from AB183 infection after treatment by simultaneous lysAB- vT2 fusion endolysin and antimicrobials; CuCl_2_ (1.62 mM) and colistin sulphate (0.5 µg/ml).

For the cytotoxicity assays, Basal LDH Activity seemed to be relatively high at around 40%. LysAB-vT2-fusion alone seemed to lead to an approximate 10% increase in LDH release from T24 human cells compared to the basal condition. This was returned as a statistically significant difference relative to the untreated group (*; *p* < 0.05). Co-incubation of lysAB-vT2-fusion with T24 human cells infected with AB183 led to a partial reduction of LDH release from T24s (~ 15% decrease compared to cells with AB183 and empty vehicle added), which indicated that the endolysin was capable of inactivating AB183 at a 2 mg/ml concentration, however relative cytotoxicity levels remained high (~ 75%) (Fig. 8A).

To assess the potential for use of endolysins with other antimicrobials, the cytotoxicity of co-treatment of infected and non-infected human epithelial cells with lysAB-vT2 fusion and the antimicrobials: copper chloride (CuCl_2_) and colistin sulphate. Treatment of AB183 infected and uninfected T24 human epithelial cells by lysAB-vT2 (2 mg/ml) and copper chloride (1.62 mM) yielded no significant difference in LDH release compared to untreated/uninfected control. The same was found with the exposure of T24 cells to lysAB-vT2 fusion (2 mg/ml) and colistin sulphate (0.5 µg/ml), with no difference in cytotoxicity to T24 cells compared to spontaneous release controls. AB183 infection of T24 cells was also successfully treated in all antimicrobial treatment combinations (Fig. 8B).

## Discussion

Bacteriophage derived enzymes such as endolysins have been studied to combat the emergence of multidrug-resistant bacteria. In this study, we investigated the endolysin protein from vPhT2.

The diversity of endolysins depends on the enzymatic catalytic domain (ECD) responsible for cleaving a specific peptidoglycan bond and on cell binding domains (CBD), which are responsible for recognizing specific epitopes on the bacterial cell wall^18^. Endolysins with a cell wall binding domain (CBD) has been identified only in some *A. baumannii* bacteriophages which were endolysins ElyA1 and ElyA2 that contained a peptidoglycan binding domain PG3 at the N-terminal end (11). BLAST analysis of lysAB-vT2 predicted one ECD and a lysozyme conserved domain, but a CBD was absent in this endolysin (Fig. 1A). The ECD of endolysins lysAB-vT2 were a muramidase (N-acetyl-*β*-D-muramidase) that cleaves *β*-1, 4-glycosidic bonds between N-acetylmuramic acid and a N-acetylglucosamine, which was in agreement with previous reports^7,8^. Phylogenetic relationship studies between lysAB-vT2 with other *A. baumannii* lysins showed that its closely related to an endolysin from unclassified phage RL_2015 and phage AM24 isolated in 2014 (Moscow, Russia)^19^.

Two recombinant endolysins were expressed and purified. In agreement with a previous report, we found that both endolysins showed the board spectrum to lyse autoclaved cells of Gram-negative bacteria^8^. The MIC of native lysAB-vT2 was higher than 10 mg/ml. Due to the lack of CBD of native lysAB-vT2, the native endolysin cannot pass through and reach the inner peptidoglycans layer in the periplasmic space of Gram-negative bacteria^17^. In addition, we found a holin lysis mediator (GenBank: QHJ75701.1) in the genome of vPhT2. Holins are phage-encoded membrane proteins that control access of phage-encoded endolysins to the peptidoglycan and thereby trigger the lysis process. The physicochemical properties of Gram-negative lysins, such as net charge, hydrophobicity, hydrophobic moment and aliphatic index are responsible for an improved interaction with Gram- negative envelope^20^. Thus, we generated hydrophobic amino acid fusion with endolysin of lysAB- vT2, since hydrophobic polypeptides had enabled penetration of the outer membrane^21^. Compared to native lysAB-vT2, the MIC of lysAB-vT2-fusion was decreased to 5 times and we have focused on the activity of lysAB-vT2-fusion. The lipid part at the C-terminal of lysAB-vT2-fusion helped anchor the antibiotic to the bacteria membrane through its lipid part as report by Anikin et al.^22^.

The combinations of colistin and endolysin against clinical strains of multi-drug resistant bacteria, such as *A. baumannii* and *P. aeruginosa* have been reported in vitro and in vivo ^8,11^. Our study found that the interactions between lysAB-vT2-fusion had elevated antibacterial activity with colistin, polymyxin B, and CuCl_2_. Colistin and polymyxin B are positively charged antibiotics that bind through the negatively charged phosphate groups of lipid A and its hydrophobic terminal acyl fat chain, causing an expansion of the external outer membrane (OM)^23^. A permeabilization of the OM occurs, allowing endolysin to get through the OM and cleave the hydrolyzing of the peptidoglycan layer. There was no difference in the combination of lysAB-vT2-fusion with imipenem at the 0.25 × MIC level of both agents. Imipenem, a beta-lactam antibiotic of the carbapenems class, acts by inactivating the penicillin-binding proteins (PBPs). At the sub-MIC level, both agents are limited in their to access the peptidoglycan due to the presence of bacterial outer membrane as a structural barrier. In addition, copper (Cu^+^) ions have been used as antimicrobial agents in agriculture and health care. At sub-MIC levels, the cells exposed to copper surfaces can induce membrane damage^24^ and allowing endolysin to get through the OM to enhanced antibacterial activity of the enzyme. The lysAB-vT2-fusion plus colistin can inhibit the growth of various strains of *A. baumannii*, such as XDRAB, MDRAB, but not the colistin resistant *A. baumannii* (COLRAB). This phenomenon can be explained by the fact that MIC of COLRAB in this study was 32 ug/ml and a low concentration of colistin (0.5 µg/ml) was used, in combination with enzyme and this concentration may not be enough to destroy the external outer membrane (OM) of the colistin resistant strains and does not permit the enzyme to function.

In order to apply lysAB-vT2-fusion as an antibacterial product in the clinical environment, thermal stability, biofilm assays and cytotoxicity of lysAB-vT2-fusion were determined. Thermal stability tests showed that the endolysin still functions at a broad range of temperature from 4 to 60 °C. However, at 80 °C, the percent inhibition of lysAB-vT2-fusion dropped due to protein denaturation. In agreement with previous reports, lysAB-vT2-fusion can inhibit biofilm formation and decreases mature biofilms^10,25,26^. Our study found that 4 mg/ml endolysin and phage vPhT2 can inhibit mature biofilms. The MBEC concentration was higher than the MIC level. This phenomenon can be explained by the structure of the biofilms that are encased by an extracellular polymeric substance (EPS) matrix^26^ and contribute to high MBEC. It was found that lysAB-vT2 fusion can be effectively used in conjunction with phage therapy in disrupting *A. baumanni* biofilms. Contrary to findings in literature that find staggered antimicrobial-phage therapy to be more effective, the simultaneous treatment using both phage and lysAB-vT2 fusion endolysin at the same time was found to be more effective than sequential treatment in disrupting biofilms^27^. This occurance may be explained that unlike more traditional antimicrobials, phage endolysins do not interfere with the phage life cycle within biofilms.

In contradiction to previous reports *A. baumannii* phages and phage enzymes were found to be safe and presented no cytotoxicity to human cell lines^12,16^, even when used in combination with other antimicrobials. Our study found that the endolysin is capable of inactivating *A. baumannii* to cause cell toxicity, however relative cytotoxicity levels remain high. This is due to the fact that high concentration of the enzyme was used compare to the previous study.

In conclusion, we cloned and expressed phage hydrophobic amino acid fusion endolysin, lysAB-vT2-fusion. This enzyme has antibacterial activity, has the ability to decrease mature biofilm and has synergistic effect with colistin. Combined with colistin or on its own, this enzyme can inhibit various strains of *A. baumannii*, including MDRAB, XDRAB and phage resistant *A. baumannii.*

## Materials and methods

### Bacterial strains, bacteriophage, and growth conditions

Bacteriophage vB_AbaM_PhT2 (vPhT2) (MN864865) was isolated and characterized as previously described^10^. To test the antibacterial activity, we used *A. baumannii* isolates collected from 2006 to 2021 from five hospitals in Thailand and *A. baumannii* isolated in 2021 from the hospital environment of Naresuan University hospital (Table S1). The *A. baumannii* strains were drug-sensitive *A. baumannii* (NR), multidrug-resistant *A. baumannii* (MDRAB), extremely drug- resistant *A. baumannii* (XDRAB), carbapenem-resistant *A. baumannii* (CRAB), colistin resistant *A. baumannii* (COLRAB), and phage resistant *A. baumannii* strains (PRAB) from a previous study^28^. Antibiotic susceptibility of all isolates was determined according to CLSI 2017^29^ and is shown in Table S1. *A. baumannii* strains were grown in Luria–Bertani (LB) broth or agar. Recombinant *E. coli* BL21 (DE3) containing the constructs pET28a-lysAB- vT2 and pET28a- lysAB-vT2-fusion were grown in Luria–Bertani (LB) broth and agar, supplemented with 50 µg/ml of kanamycin and 10 ug/ml of chloramphenicol. The protocol was approved by the Naresuan University Institutional Biosafety Committee, and the project number was NUIBC MI 64-08-32.

### Cloning, expression and purification of recombinant phage-derived endolysin (lysAB-vT2) and phage endolysin hydrophobic fusion (lysAB-vT2-fusion)

Endolysin gene of vPhT2 (Accession number MN864865) flanked by the restriction sites *BamH*I and *EcoR*I in pET28a was generated using gene synthesis system (gene script). In addition, the endolysin hydrophobic fusion was generated in pET28a, by adding a gene encoding hydrophobic amino acids (F-I-L-I-V-F-V-L-I-I-A-P) at the C-terminal. The pET28a vectors that contained the endolysin gene (lysAB-vT2) and the endolysin hydrophobic fusion (lysAB-vT2- fusion), were transformed in *E. coli* DH5*α* and the plasmids were purified and subcloned into *E. coli* BL21 (DE3) pLysS. Restriction digestion was used to verify the integrity of the cloned fragment. For overexpression of recombinant phage enzymes, a log phase culture of *E. coli* BL21 (DE3) pLysS containing pET28a with lysAB- vT2 or lysAB- vT2-fusion (A600–0.5) was induced by the addition of IPTG to the final concentration of 1 mM. After incubation for 4 h at 37 °C, cells were pelleted, washed and frozen at − 80 °C. The frozen cells were resuspended in 10 ml lysis buffer (145 mM NaCl, 20 mM Tris–HCl, pH 7.4). The samples were freeze–thaw 3–4 times and then 100 µl Triton X-100 (final conc. 1% v/v) was added and sonicated on ice until clear lysate was observed. After that, samples were centrifuged at 12,000 rpm at 4 °C for 10 min. Supernatants (soluble fraction) were collected to purify the recombinant proteins using His-tag affinity chromatography column (His·Bind Kits, Novagen, Germany) according to the manufacturer’s instructions. The purified protein fractions were pooled and dialyzed against dialysis buffer overnight. The purity of protein was checked by 12% SDS-PAGE, followed by staining of the SDS-PAGE gels with Coomassie brilliant blue G-250.

### Plate lysis assay

Plate lysis assays were performed as described previously^7^ using bacterial cells of *A. baumannii* ATCC 19606, *A. baumannii* AB003 (MDRAB), *A. baumannii* AB329 (XDRAB), *Escherichia coli* ATCC 25922, *Pseudomonas aeruginosa* ATCC 27853, *Klebsiella pneumoniae* ATCC 27736 or *Staphylococcus aureus* ATCC 6538. Bacteria were grown to mid log phase, collected, washed once and suspended in PBS. The suspension was then autoclaved and centrifuged. The resulting pellet was resuspended in PBS (2% [vol/vol] of initial culture volume) and was used as the substrate. 5 µl of purified enzymes (4 mg/ml) were spotted onto the agar plate (1.5%) containing the substrate (5%). An equal volume of 20 ug egg white lysozyme (EWL) was used as control. The spotted plates were incubated at room temperature. Clear zones were observed and diameter of clear zones were measured.

### Minimum inhibitory concentration (MIC)

The MIC of lysAB-vT2 or lysAB-vT2-fusion against *A. baumannii* ATCC 19606 and *A. baumannii* clinical isolates was determined according to the standard broth microdilution method of the Clinical and Laboratory Standards Institute^24^. Briefly, two-fold serial dilutions of each enzyme were prepared in Mueller Hinton broth (MHB) in a sterile 96-well U-bottom microtiter plate. Inoculum was prepared by culturing *A. baumannii* on LBA and was kept at 37 °C overnight. Colonies of *A. baumannii* were resuspended in 0.85% NaCl solution at an inoculum concentration of 0.5 McFarland using densitometer to achieve a concentration of 1 × 10^5^ CFU/ml. Each well was inoculated with 50 µl of bacterial solution, so that the final concentration of the inoculum was 0.5 × 10^5^ CFU/well. The plate was incubated at 37 °C overnight. MIC was defined as the lowest concentration of enzymes that inhibited the visible growth.

### Synergism of lysAB-vT2-fusion with antibiotics, disinfectants, heavy metals, and bacteriophage

The interactions between lysAB-vT2 or lysAB-vT2-fusion with three antibiotics (imipenem, colistin and polymyxin B), three disinfectants (quaternary ammonium compound (QACs), Chloroxylenol, Sodium Hypochlorite), two heavy metals (CuCl_2_, ZnCl_2_) and bacteriophage vPhT2 were screened by growth inhibition assay at 0.25 × the MIC of two agents. The host for testing of all agents was ATCC 19606. *A. baumannii* AB329 was used for testing bacteriophage. The percent inhibition was calculated as the formular below

$$\% {\text{ inhibition }} = \frac{{{\text{the}}\;{\text{absorbance}}\;{\text{of}}\;{\text{controls}} - {\text{the}}\;{\text{absorbance}}\;{\text{of}}\;{\text{treated}}\;{\text{wells}} \times {1}00}}{{{\text{the}}\;{\text{absorbance}}\;{\text{of}}\;{\text{control}}}}$$

The checkerboard broth microdilution method was used to determine interaction between lysAB-vT2-fusion with the selected antibiotics^30^. Test results of two agents acting individually or in combination were used to calculate FIC index (FICI) which was the sum of the fractional inhibitory concentrations (FICs) of two agents as the formula: FIC index = FIC of lysAB-vT2-fusion + FIC of antibiotic, where FIC lysAB-vT2-fusion is the MIC of lysAB-vT2-fusion in the combination/MIC of lysAB-vT2-fusion alone and FIC antibiotic is the MIC of antibiotic in the combination/MIC of antibiotics alone.

FIC index of enhanced agents was interpreted as follows: FIC ≤ 0.5, synergistic; FICI = 0.5–4, additive or indifference; and FICI > 4, antagonistic^25^.

### Antibacterial activity of lysAB-vT2-fusion and lysAB-vT2-fusion plus colistin

Antibacterial activity of lysAB- vT2-fusion against *E. coli* ATCC 25922, *P. aeruginosa* ATCC 27853, *K. pneumoniae* ATCC 27736 and various strains of *A. baumannii*, which were drug-sensitive strain (NR), multidrug-resistant *A. baumannii* (MDRAB), extremely drug-resistant *A. baumannii* (XDRAB), carbapenem-resistant *A. baumannii* (CRAB) and colistin resistant *A. baumannii* (COLRAB) was tested. Briefly, *A. baumannii* were cultured in LB agar incubated overnight at 37 °C. Then, cultures were diluted into LB broth, the turbidity of cell suspensions were adjusted to an equivalent 0.5 McFarland standard as measured by densitometer corresponding to approximately 1 × 10^8^ CFU/ml. The adjusted cell suspensions were diluted 1:100 in double strengthen Mueller Hilton broth and 50 µl was inoculated into 96-well microtiter plates wells U bottom (Nunc,USA). 50 µl of the enzymes at the MIC concentration (2 mg/ml) or enzymes (10 µg/ml) with colistin (0.5 µg/ml) at sub-MIC concentrations were added to each well. After overnight incubation at 37 °C, 50 ul of 0.1% filter sterilized TTC (Hi-media) was added. The plate was incubated at room temperature in the dark for 3 h. The absorbance at OD_540_ was measured using a Synergy 2 multi-mode microplate reader (BioTek Instruments, Winooski, VT, USA). The experiment was replicated twice with triplicate samples. The percentage inhibition against all *A. baumannii* was calculated as previously described^7^.

### Thermal stability

To determine the stability of antibacterial activity under various temperature conditions, 2 mg/ml of lysAB-vT2-fusion was incubated at 4, 20, 40, 60 and 80 °C, each for 30 min. After incubation, antibacterial activity was determined using bacterial inhibition assay. Protein concentration was measured after thermal treatment and percent protein reduction was calculated.

### Biofilm assay of lysAB-vT2-fusion and cytotoxicity of lysAB-vT2-fusion

For the biofilm assay, the endolysin and phage vPhT2 were applied to 72-h *A. baumannii* AB183 biofilm producing strain and the purified endolysin’s ability to clear 72-h biofilms was assessed via the Minimum Biofilm Eradication Concentration assay (MBEC assay). Biofilms were grown over 72 h in LB broth at 37 °C, were exposed to varying concentrations of purified endolysin and to 1 × 10^8^ PFU/ml of phage vPhT2 in Mueller Hinton Broth for 24 h. Endolysin-phage adjuvant assays was performed with treatment of 72 h mature AB183 biofilms with phage and endolysin simultaneously and sequentially, with sequential treatment first treating biofilms for 24 h with phage vPhT2 and then subsequent 24 h treatment with lysAB-vT2 fusion endolysin. After treatment, biofilms were sonicated off into PBS and the number of surviving bacteria in the biofilm were quantified by plating the resulting PBS containing sonicated biofilm to determine CFU. For the cytotoxicity assay, human T24 urinary bladder epithelial cells (ATCC® HTB-4™) and *A. baumannii* AB183 were used and prepared as previously described (Styles 2020). LDH cytotoxicity assay performed using Invitrogen CyQUANT kit according to manufacturer’s protocol.

### Statistical analyses

All tests were independently performed in triplicate, and the data were presented as mean ± standard deviation. Data analysis was performed using one way ANOVA for group comparisons to control and unpaired T test for pairwise comparisons (Fig. 7A). *p* < 0.05 was considered statistically significant.

## Supplementary Information

Supplementary Information.

## Data Availability

Datasets for this research are included in the supplemental tables.
